# Clinical efficacy and safety of sequential accelerated theta burst stimulation for suicidal ideation in adults with major depressive disorder: study protocol of a randomized controlled trial

**DOI:** 10.3389/fpsyt.2026.1795183

**Published:** 2026-03-31

**Authors:** Haobo Leng, Guilan Huang, Zimeng Yang, Lu Sun, Guofu Zhang, Caili Ren

**Affiliations:** 1 Department of Psychiatry, The Affiliated Mental Health Center of Jiangnan University, Wuxi, Jiangsu, China; 2 Department of Rehabilitation Medicine, The Affiliated Mental Health Center of Jiangnan University, Wuxi Central Rehabilitation Hospital, Wuxi, Jiangsu, China

**Keywords:** DLPFC (dorsolateral prefrontal cortex), MDD (major depressive disorder), neuromodulation, suicidal ideation, TBS (theta burst stimulation)

## Abstract

**Introduction:**

Major depressive disorder (MDD) is a prevalent psychiatric condition associated with significant suicide risk. Sequential accelerated theta-burst stimulation (aTBS), which integrates time-efficient stimulation with sequential modulation of multiple targets, represents a promising neuromodulation strategy. However, the efficacy and safety of sequential aTBS in adults with MDD and active suicidal ideation remain unexplored. This study aims to evaluate the therapeutic safety and effect of sequential aTBS on suicidal ideation in adults with MDD, and to explore its associated neurophysiological mechanisms using electroencephalography-derived P300 event-related potentials.

**Methods and analysis:**

This study is a single-blind, randomized controlled trial. Fifty-six adults with MDD will be recruited and randomly assigned (1:1) to receive either active sequential bilateral Dorsolateral Prefrontal Cortex (DLPFC) aTBS (10 weekday sessions; 3600 pulses per session) consisting of continuous theta-burst stimulation (cTBS) applied to the right DLPFC followed by intermittent theta-burst stimulation (iTBS) applied to the left DLPFC, or sham stimulation. The primary outcomes will be response and remission rates based on the 17-item Hamilton Depression Rating Scale (HAMD-17). Secondary outcomes will include the Beck Depression Inventory-II (BDI-II), the Columbia Suicide Severity Rating Scale (C-SSRS), and electroencephalography (EEG)-derived electrophysiological markers of cognitive processing (P300). Safety and tolerability will be systematically evaluated throughout the study using the Treatment Emergent Symptom Scale (TESS) to record stimulation-related adverse events. Outcome measures will be assessed at baseline, immediately after the 10-day intervention, and at 2-week and 4-week follow-ups to evaluate the short-term sustainability of treatment effects.

**Discussion:**

The results of this study will provide information regarding the efficacy and safety of sequential aTBS for MDD, evaluating its feasibility and thereby laying a foundation for future clinical interventions and scientific research.

**Clinical Trial Registration:**

https://www.medicalresearch.org.cn/index, identifier ChiCTR2500109181.

## Introduction

Major depressive disorder (MDD) is a highly prevalent mental health condition in adults, characterized by a lifetime prevalence of 10%-15%, high relapse rates, and particularly severe suicide risk. Suicide remains one of the leading causes of mortality among adults worldwide (1). The central clinical challenge involves suicidal ideation, which affects approximately 60%-80% of adult patients (2). Despite its severity, currently available pharmacological and psychotherapeutic treatments remain limited in their ability to provide rapid stabilization. Antidepressant medications require weeks to exert therapeutic effects, making them unsuitable for acute risk management, while high relapse rates frequently lead to the re-emergence of suicidal ideation even after initial improvement (3, 4). Therefore, there is an urgent need for rapid-acting, safe, and evidence-based interventions specifically targeting suicidal ideation in adults with MDD.

Transcranial magnetic stimulation (TMS) is an FDA-approved intervention for depression, valued for its non-invasiveness, favorable safety profile, and clinical utility (5). The conventional 10-Hz left Dorsolateral Prefrontal Cortex (DLPFC) protocol, however, requires a 6-week course and yields modest response and remission rates (29%–46% and 18%–32%), with treatment burden contributing to substantial dropout (6, 7). These limitations have accelerated the search for more time-efficient neuromodulation strategies. Theta-burst stimulation (TBS) offers a condensed, time-efficient alternative: intermittent theta-burst stimulation (iTBS) increases cortical excitability when applied to the left DLPFC, whereas continuous theta-burst stimulation (cTBS) suppresses hyperactivity in the right DLPFC, with both forms capable of inducing rapid neuroplastic changes (8–10). The 2022 FDA clearance of the Stanford Accelerated Intelligent Neuromodulation Therapy (SAINT) protocol—which consists of 10 daily sessions, each delivering 1,800 pulses with a 50-minute inter-session interval over five consecutive days, totaling 90,000 pulses, and is characterized by individualized functional-connectivity targeting and high-dose, short-interval stimulation—highlighted the clinical feasibility of accelerated theta-burst stimulation (aTBS) paradigms (11). Subsequent clinical studies have demonstrated that SAINT produces rapid antidepressant effects and high remission rates in treatment-resistant depression (12). In addition, recent studies have shown that accelerated cTBS and iTBS interventions can effectively alleviate suicidal ideation in patients with treatment-resistant depression (13). An independent study focusing on iTBS also confirmed that this therapy significantly improved suicidal ideation among such patients (14). Nevertheless, existing aTBS paradigms remain largely restricted to single-target stimulation, leaving critical questions about stimulation sequences and network-level modulation unresolved.

Based on these considerations, sequential aTBS has emerged as a novel TMS strategy. It integrates high-intensity, short-interval theta-burst stimulation with sequential modulation of multiple cortical targets. This design enables a single treatment session to produce synergistic excitatory and inhibitory effects, forming an “excitation-inhibition-regulation” circuit that may enhance the plasticity of large-scale brain networks (15). Compared to the SAINT protocol, sequential aTBS is designed to reduce per-session stimulation intensity and overall treatment burden by distributing neuromodulatory effects across multiple cortical targets. By stimulating multiple functionally connected brain regions—typically the excitatory left DLPFC and the inhibitory right DLPFC—it promotes interhemispheric balance and accelerates emotional regulation. Theoretically, this multi-target approach aligns with the contemporary understanding of “depression as a network disorder” as MDD pathogenesis is associated with dysfunction in the prefrontal-limbic circuitry (16).

Current evidence regarding sequential accelerated theta-burst stimulation (aTBS) for the treatment of depression remains limited. Existing studies have primarily involved adults with treatment-resistant depression, including naturalistic observational studies of bilateral sequential aTBS targeting the dorsolateral prefrontal cortex (15), or have been confined to protocol designs for adolescents with major depressive disorder accompanied by suicidal ideation (17). Notably, these studies are characterized by heterogeneous clinical endpoints and involve populations such as adolescents, highly treatment-refractory individuals, or mixed groups with various depressive disorders. To date, no randomized controlled trial has specifically investigated sequential bilateral aTBS in adults with MDD who present active suicidal ideation.

Growing evidence suggests that suicidal ideation in MDD patients is associated with impairments in cognitive control, attentional regulation, and emotional processing, primarily involving the prefrontal-limbic circuitry (18, 19). Investigating these neurophysiological mechanisms may provide objective biomarkers for identifying suicide risk and evaluating treatment efficacy. Electroencephalography (EEG), with its high temporal resolution, enables direct assessment of cortical excitability and cognitive dynamics under neuromodulation. Among EEG-derived event-related potentials, the P300 component—typically elicited by an auditory oddball paradigm—serves as a reliable indicator of attentional resource allocation and information processing (20, 21). Consistent evidence shows reduced P300 amplitude and prolonged latency in depressed patients, reflecting diminished cognitive efficiency and prefrontal dysfunction (22). Moreover, effective antidepressant interventions—including high-frequency repetitive transcranial magnetic stimulation (rTMS)—have been shown to normalize P300 parameters in parallel with clinical improvement (23). These findings support the use of P300 as a sensitive electrophysiological marker of treatment response. Therefore, evaluating P300 changes before and after sequential aTBS may provide crucial insights into the neurophysiological mechanisms underlying its therapeutic effects, thereby helping to elucidate the intervention’s neurophysiological principles of action.

### Objectives

The primary objective of this randomized controlled trial is to evaluate the clinical efficacy of sequential accelerated Theta Burst Stimulation (aTBS) in adults with MDD. Our central hypothesis is that, compared to sham stimulation, sequential aTBS will induce a faster and more significant alleviation of depressive symptoms, with this therapeutic effect remaining significant throughout the subsequent 2-week and 4-week follow-up periods. A secondary objective of this study is to systematically evaluate the safety, tolerability, and adverse event profile of sequential aTBS in the target population. For this safety assessment, the Treatment Emergent Symptom Scale (TESS) will be administered to capture and characterize common adverse events, including headache, dizziness, nausea, scalp discomfort, and muscle twitching. For mechanistic exploration, this study will employ high-temporal-resolution EEG to capture the P300 event-related potential as a neurophysiological indicator, aiming to elucidate the mechanisms by which aTBS modulates cortical excitability and cognitive-emotional processing, thereby identifying potential biomarkers for future treatment strategies.

## Materials and methods

### Study design

This randomized, sham-controlled clinical trial was approved by the Ethics Committee of Wuxi Mental Health Center and prospectively registered in the Chinese Clinical Trial Registry (ChiCTR; official website: www.chictr.org.cn, Registration No.: ChiCTR2500109181). All procedures will adhere to the principles of the Declaration of Helsinki and the Guidelines for Good Clinical Practice (GCP). Reporting will follow the SPIRIT 2025 recommendations, and the completed SPIRIT checklist is available in **Supplementary Material S1**.

**Figure 1:**
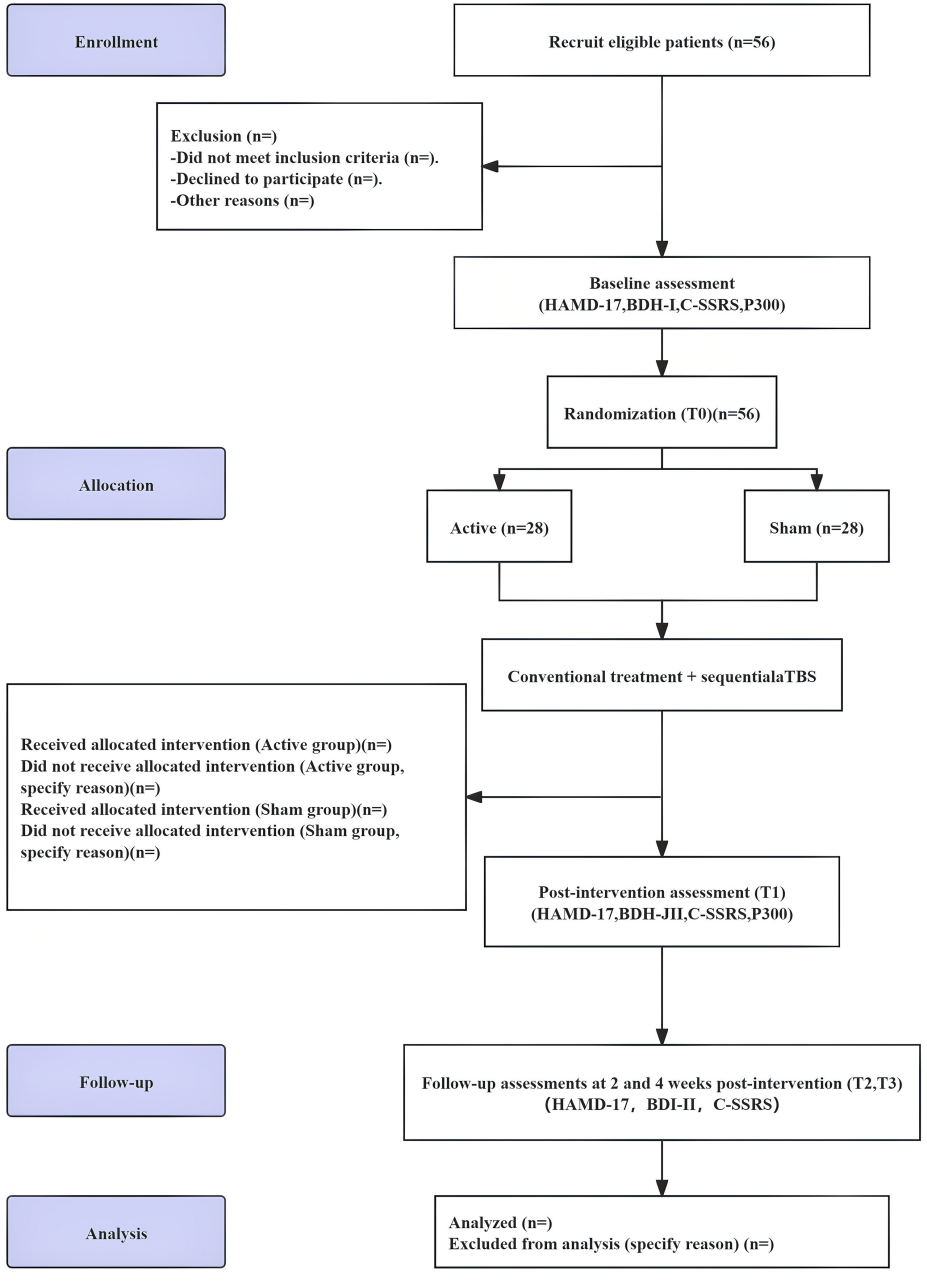
CONSORT flow diagram of the study protocol; Patient screening; Depressed patients (n=56); Randomized allocation; Active stimulation group (n=28); Sham stimulation group (n=28); T0, baseline (randomization); T1, post-intervention; T2, 2-week follow-up; T3, 4-week follow-up; HAMD-17, 17-item Hamilton Depression Rating Scale; BDI-II, Beck Depression Inventory-II; C-SSRS, Columbia-Suicide Severity Rating Scale; aTBS, accelerated theta-burst stimulation.

This trial will be conducted at Wuxi Mental Health Center between September 2025 and August 2027. The assessment and screening will be conducted by two practicing physicians from Wuxi Mental Health Center. Eligible participants will be randomly allocated (1:1) to receive either active sequential accelerated theta burst stimulation (aTBS; 3,600 pulses per session, 10 sessions total) or sham stimulation. Outcome measures will include the 17-item Hamilton Depression Rating Scale (HAMD-17) (24), Beck Depression Inventory (BDI-II) (25), Columbia Suicide Severity Rating Scale (C-SSRS) (26), Treatment Emergent Symptom Scale (TESS) (27) and event-related potential P300. All assessments will be administered at baseline (T0), immediately after the final stimulation session (T1), and at 2-week (T2) and 4-week (T3) follow-ups. A detailed flow diagram of the trial is shown in **Figure 1**, and the trial schedule is presented in **Table 1**.

**Table 1:** Detailed trial schedule.

	Study period							
	Enrollment	Allocation	Post-allocation				Follow-up 1	Follow-up2
Time point	Day-1	Day 0	AFT	Day1	~	Day 10 (Intervention period)	Week2	Week4
Enrollment:								
Eligibility screen	×							
Informed consent	×							
Randomization	×							
Allocation		×						
Interventions:								
Conventional treatment + sequential aTBS			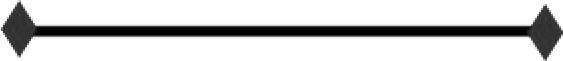					
Conventional treatment + sham sequential aTBS			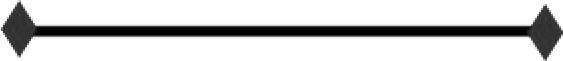					
Assessments:								
HAMD-17		×				×	×	×
BDI-II		×				×	×	×
C-SSRS		×				×	×	×
P300		×				×		
Case report forms	×		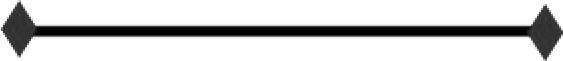					
Adverse events			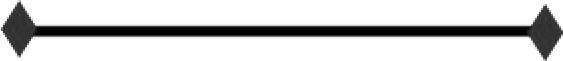					
TESS			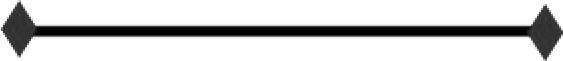					
Blinding			×					
TASS	×							
ATHF	×							

AFT; Immediately post-allocation (after randomization and before the first intervention); HAMD-17, 17-item Hamilton Depression Rating Scale; BDI-II, Beck Depression Inventory-II; C-SSRS, Columbia-Suicide Severity Rating Scale; P300, Electroencephalography; ATHF, Antidepressant Treatment History Form; TASS, Transcranial Magnetic Stimulation Safety Screen; TESS, Treatment Emergent Symptom Scale ×, occurs at that time.

### Patient recruitment

Patient recruitment follows a four-step procedure;

Step 1: Promotion for inpatient recruitment will be conducted through outpatient clinics, among inpatients, and at surrounding community service centers.

Step 2: A clinical research assistant will assess potential participants for eligibility according to the predefined inclusion and exclusion criteria.

Step 3: The study coordinator will schedule an interview with eligible individuals to provide detailed information about the study objectives, procedures, potential risks and benefits.

Step 4: Participants who fully understand the study information and voluntarily agree to participate will provide written informed consent. The written informed consent form is provided in **Supplementary Material S2**.

### Inclusion and exclusion criteria

Inclusion criteria are as follows; meeting the diagnostic criteria for depression as defined by the Diagnostic and Statistical Manual of Mental Disorders, Fifth Edition (DSM-5) (28), or having been taking the same antidepressant for at least two weeks; being Han Chinese residents aged 18 to 60 years; having at least a primary school education; voluntarily agreeing to participate; having a HAMD-17 score ≥17; and having a C-SSRS suicidal ideation score ≥3. Exclusion criteria include meeting the DSM-5 diagnostic criteria for other psychiatric disorders such as schizophrenia or bipolar disorder; being comorbid with poorly controlled severe physical diseases, such as coronary heart disease or thyroid dysfunction; having a history of traumatic brain injury with loss of consciousness or epilepsy; having any severe physical illness requiring long-term medication and/or a history of substance abuse; being pregnant, lactating, or planning pregnant; and having a recent history of fever, acute infection, or contagious diseases. A detailed list of the inclusion and exclusion criteria is presented in **Table 2**.

**Table 2:** Method of ascertainment and justification for inclusion and exclusion criteria.

Inclusion criterion	Method of ascertainment	Justification
Meet the first-episode diagnostic criteria for depression as defined by the Diagnostic and Statistical Manual of Mental Disorders, Fifth Edition (DSM-5), or have been taking the same antidepressant for at least two weeks	Medical records,HAMD-17,BDI-II	Determine the target population of the experiment and medication records
Han Chinese residents aged 18 to 60 years, regardless of gender	Medical records	The population with depression is mainly concentrated in this age group and is suitable for experimental interventions
Educational level of primary school or higher	Directly ask the patient or their family	Be able to cooperate with the intervention and assessment of the experiment
Voluntary participation in the study	Meet with the principal investigator to discuss the study and sign the informed consent form	Required
A score of ≥17 on the 17-item Hamilton Depression Rating Scale (HAMD-17)	The higher the score, the more severe the depressive symptoms are, and a score of ≥17 usually corresponds to “moderate or more severe depression”	Target population of the trial
A score of ≥3 on the Columbia Suicide Severity Rating Scale (C-SSRS) for suicidal ideation	A score of ≥3 on the suicidal ideation dimension indicates the presence of suicidal thoughts with a certain intensity or frequency	Target population of the trial
Exclusion Criteria	Method of ascertainment	Justification
Meet diagnostic criteria for other mental disorders as defined by the DSM-5, such as schizophrenia or bipolar disorder	Medical records, outpatient physician diagnosis	Exclude interference of other diseases on the study
Comorbid with poorly controlled severe physical diseases, such as coronary heart disease or thyroid dysfunction	Medical records, clinical examinations	The study may exacerbate other physical diseases
A history of craniocerebral trauma resulting in loss of consciousness, or a history of epilepsy	Medical records, clinical examinations	Contraindications to transcranial magnetic stimulation (TMS)
Any severe physical illness requiring chronic medication; and/or a history of substance abuse/dependence	Medical records, clinical examinations	The study may exacerbate other physical diseases
Women who are pregnant, lactating, or planning to conceive	Medical records, clinical inquiry	Contraindications to transcranial magnetic stimulation (TMS)
Recent history of fever, acute infection, or contagious diseases	Medical records, clinical examinations	Protect the safety of participants and researchers, and avoid interfering with the accuracy of study results

DSM-5, Diagnostic and Statistical Manual of Mental Disorders, Fifth Edition; TMS, Transcranial Magnetic Stimulation.

### Randomization and allocation concealment procedure

Computer-generated random allocation sequence (1:1 ratio) will be created using statistical software (SAS) by an independent individual who is not involved in participant recruitment, thereby ensuring that investigators cannot access or influence the original sequence. Allocation concealment will be implemented using opaque, sealed kraft paper envelopes that prevent visualization of contents under light. Each envelope will be labeled with a unique participant number corresponding to the randomization sequence but will not contain any indication of group assignment on the exterior. The printed group assignment will be folded, placed inside the appropriately numbered envelope, and sealed. An additional transparent outer sleeve (marked as “Unopened”) will be applied over the envelope, ensuring integrity through double sealing. Once a participant meets all eligibility criteria and signs the informed consent form, the researcher will provide the participant’s basic information to the coordinator and request the corresponding sequentially numbered envelope. After verifying the information, the coordinator will take out the corresponding envelope in numerical order. The envelope will be opened on-site by the researcher, and the intervention will be implemented according to the grouping information inside. After opening, the date, time, and signature of the person who opened the envelope must be marked on the envelope, which will then be archived for documentation.

### Blinding

This study employs a single−blind design, in which participants will remain blinded to their treatment allocation (active or sham stimulation), while investigators administering the interventions and assessing outcomes will not be blinded due to the technical requirements of transcranial magnetic stimulation (TMS). Blinding of participants is critical to minimize expectancy effects and subjective reporting biases. Unblinding will be permitted only in cases of serious adverse events where knowledge of treatment allocation is required for clinical management. In all other circumstances, the blinding will be maintained until database lock and completion of the primary analysis.

### aTBS protocol

Transcranial magnetic stimulation is delivered using the Magneuro system (Nanjing Vishee Medical Technology Co., Ltd.) equipped with a figure-of-eight coil (**Table 3**). DLPFC is localized using the Beam F3 method (online calculator available at https://clinicalresearcher.org/F3/calculate.php) (29).

**Table 3:** List of TMS and EEG equipment.

Equipment category	Specific requirements	Purpose
aTBS Stimulation Device	Transcranial Magnetic Stimulator (MagVenture), equipped with a “figure-of-eight” stimulation coil (70mm in diameter), supporting parameter setting for the aTBS mode (frequency: 50Hz, inter-burst interval: 200ms)	Deliver precise sequential aTBS stimulation targeting the target brain region (left/right DLPFC)
ERP Acquisition System	64-Channel EEG Recorder (Brain Products ActiChamp), with a sampling rate of ≥1000Hz and a bandpass filter of 0.1-30Hz; Closed-Back Noise-Canceling Headphones (Sennheiser HD 280 Pro)	Record the P300 component evoked by the auditory oddball paradigm, while reducing environmental noise and electromagnetic interference
Stimulus Presentation Software	E-Prime 3.0, which supports the generation of auditory stimulus sequences (standard stimuli + target stimuli), synchronous triggering of EEG markers, and response recording	Control the parameters of the oddball paradigm to ensure that the stimulus timing is synchronized with EEG acquisition (error < 1 ms)
Auxiliary Equipment	International 10–20 System Electrode Cap, Non-Irritating Conductive Paste, Alcohol Wipes, Motor Threshold (MT) Measurement Tool, HAMD-17 Rating Scale	Reduce EEG impedance, determine aTBS stimulation intensity, and assess the severity of depressive symptoms

aTBS, accelerated Theta Burst Stimulation; DLPFC, Dorsolateral Prefrontal Cortex; ERP, event-related potential; EEG, electroencephalography.

**Figure 2:**
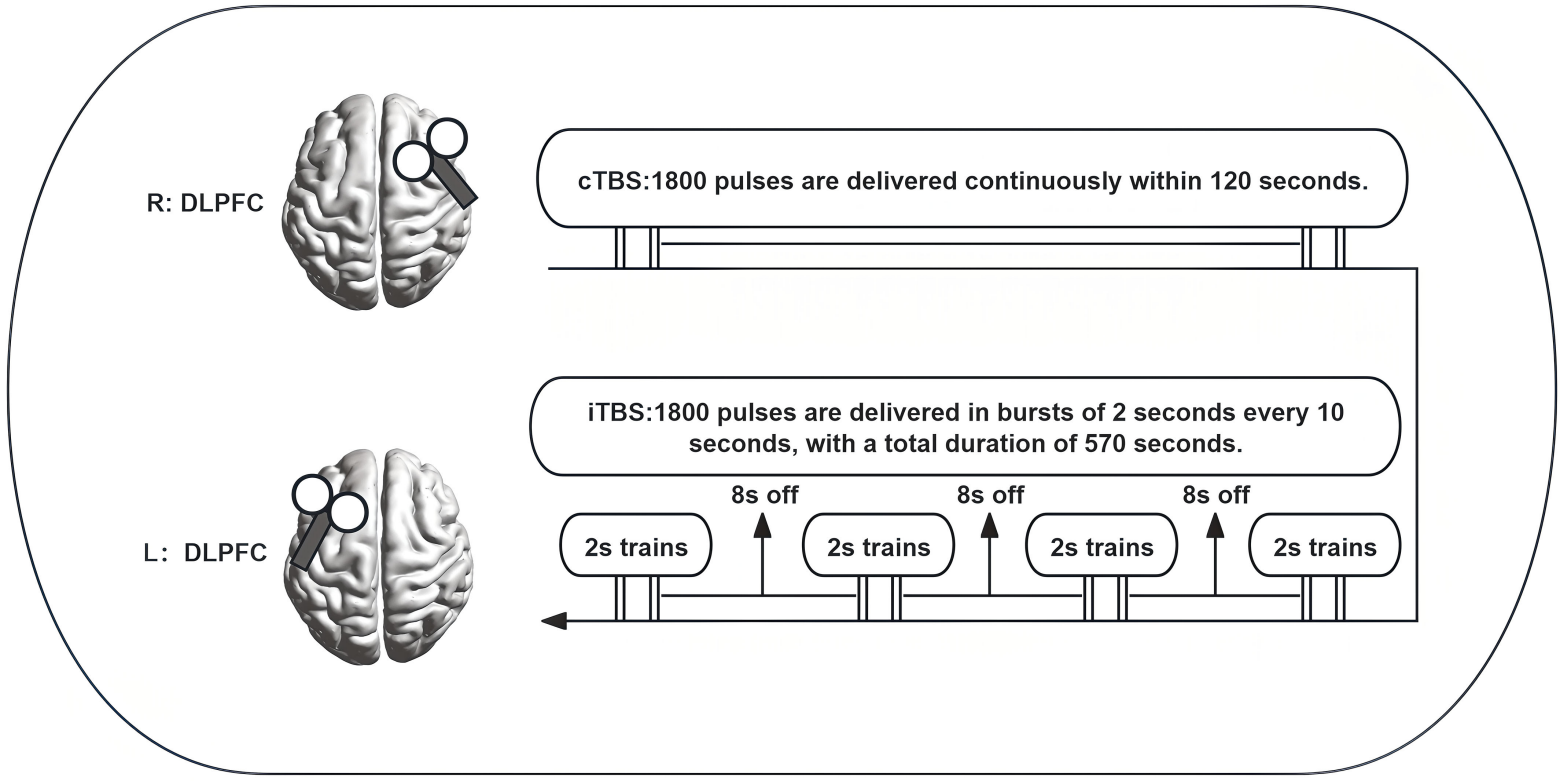
Timing and parameters of sequential aTBS intervention.

Participants in the active group receive sequential bilateral aTBS twice daily for 10 treatment days. The interval between the two daily treatment sessions in this study is set at 3 hours This interval is based on the following considerations: (1) Safety: Drawing on experience from the SAINT protocol, a 3-hour interval provides sufficient safety redundancy (12); (2) Neurophysiological recovery: This interval allows the nervous system to recover from each stimulation session, avoiding excessive cumulative effects (30); (3) Tolerability: It reduces fatigue and discomfort, thereby improving treatment adherence. The aTBS protocol, illustrated in **Figure 2**, consists of cTBS applied to the right DLPFC followed immediately by iTBS applied to the left DLPFC. Specifically, cTBS delivers 1,800 pulses over 120 seconds, whereas iTBS consists of three trains of 600 pulses (3 minutes and 9 seconds each) separated by 10-second intervals, totaling 1,800 pulses over 570 seconds. The coil is positioned tangentially to the scalp with the handle pointing posterior–laterally at approximately 45°. Sham stimulation will be delivered by tilting the coil 90° away from the scalp, while maintaining identical stimulation targets, treatment duration, and procedural protocols to those of the active intervention. This sham stimulation protocol is widely accepted and utilized in clinical trials (31).

All participants will continue to receive standardized routine clinical care—including daily psychotherapy-based skills training—administered according to a fixed protocol that will be independent of treatment allocation. If patients’ symptoms persist following these interventions, the therapist will refer them to a specialist. Detailed parameters and equipment specifications are provided in **Figure 2**; **Table 3**.

### Study outcomes

#### Primary outcome

The primary outcome is to compare the response rate of depressive symptoms between the active stimulation group and the sham stimulation group after intervention. The response rate is defined as a reduction of 50% or more in the HAMD-17 score compared with the baseline. Remission is defined as a HAMD-17 score of ≤ 7 (32).

#### Secondary clinical outcomes

The C-SSRS is a clinician-administered measure used to evaluate suicide risk by assessing both suicidal ideation and suicidal behavior. Suicidal ideation is rated on a five-level severity scale (1–5), with higher ratings reflecting more severe and imminent risk. Suicidal behavior is documented as categorical events, including preparatory actions, aborted or interrupted attempts, and actual suicide attempts. The C-SSRS has demonstrated strong reliability, validity, and predictive value in clinical populations and is widely adopted as a primary outcome measure in suicide-focused research (33).

The BDI-II is a 21-item self-report measure assessing the core symptom domains of depression. Each item is rated on a 4-point scale (0–3), yielding a total score ranging from 0 to 63, with higher scores indicating more severe depressive symptoms. The BDI-II has demonstrated excellent internal consistency (Cronbach’s α ≈ 0.94) and robust psychometric validity, supporting its use as a reliable indicator of depressive symptom severity in clinical research (34).

### Neurophysiological mechanistic outcome

#### ERP measurement

The ERP data acquisition system comprises a BrainAmp Standard amplifier (Brain Products GmbH, Germany) coupled with a 64-channel EasyCap electrode cap (Brain Products GmbH, Germany). The recording electrodes are placed at Cz and Pz, as these sites typically exhibit the largest and most stable amplitudes of the P300 component. The averaged mastoid references (A1, A2) serve as the reference electrodes, while the ground electrode is positioned at the forehead (FPz). Horizontal electrooculogram (HEOG) electrodes are placed at the outer canthi of both eyes, and vertical electrooculogram (VEOG) electrodes are positioned below the left eye. Electrode-scalp impedance is maintained below 5 kΩ for all recording sites. Electrode placement follows the 10/20 system of the International Federation of Clinical Neurophysiology, with electrode resistance < 5 kΩ (35).

### P300 data acquisition

#### Participant preparation

The experiment will be conducted in an acoustically and electrically shielded chamber with soft ambient lighting. Room temperature will be maintained at 22–25 °C to ensure participant comfort. Measures will be taken to shield monitors and cables from 50 Hz power line interference. Participants will be instructed to abstain from consuming coffee, tea, alcohol, or other central nervous system−affecting substances for 24 h prior to the experiment and to obtain adequate rest to remain alert and relaxed. Before formal data acquisition, participants will sit quietly in the laboratory for 5–10 min to adapt to the experimental environment.

#### Task instructions

The experimenter will deliver standardized, concise verbal instructions to clearly explain the auditory oddball paradigm: “You will hear two different tones through the headphones. One tone occurs frequently (the ‘high−pitch tone’), while the other occurs infrequently (the ‘low−pitch tone’). Your task is to press the response button as quickly and accurately as possible when you hear the infrequent ‘low−pitch tone’, and to make no response to the frequent ‘high−pitch tone’. Please keep your head and body still and try to minimize eye blinks throughout the recording.”

#### Stimulus presentation parameters (classic auditory oddball paradigm)

The auditory stimuli will consist a standard tone (1000 Hz pure tone, 80 % probability) and a target tone (2000 Hz pure tone, 20 % probability). Both tones will be presented binaurally at 75 dB SPL with a duration of 50–100 ms. The inter−stimulus interval will vary randomly between 1.0 and 1.5 s (36).

#### Data acquisition procedure

Before the formal experiment, participants will complete a short practice session (approximately 20–30 trials) to ensure full comprehension of the task and accurate discrimination between the two sounds. Practice data will not be included in the formal analysis.

#### Formal acquisition

**Figure 3:**
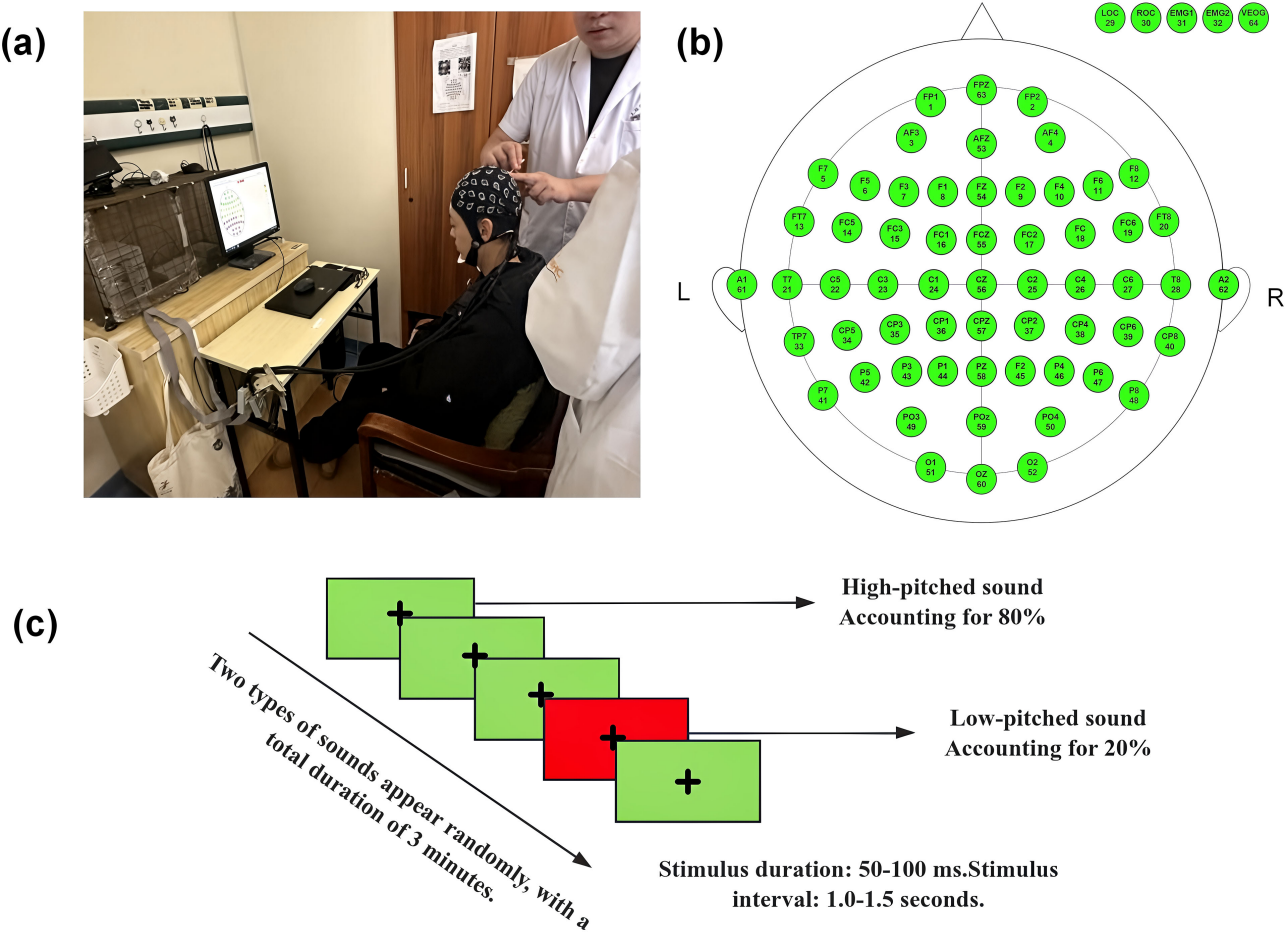
Experimental setup and paradigm for P300 assessment via EEG panel; **(a)** depicts the EEG recording setup, where a participant undergoes electrode placement with a 64-channel EEG cap, while synchronized stimulus presentation and data acquisition are managed by dedicated software. **(b)** illustrates the electrode montage following the extended 10–20 system, with “L” and “R” indicating left and right hemispheres, enabling high-resolution cortical activity monitoring. **(c)** outlines the auditory oddball paradigm used to elicit P300: two types of sounds (high-pitched, 80% probability; low-pitched, 20% probability) are presented randomly, with a stimulus duration of 50–100 ms, inter-stimulus interval of 1.0–1.5 s, and total task duration of 3 minutes. This paradigm is designed to assess attentional resource allocation and information processing efficiency, indexed by P300 latency and amplitude.

(1) EEG recording will be started. (2) Participants will sit upright in front of the screen (optionally fixating on a “+” cross−hair at the center of the screen to reduce eye movements), wear headphones, and place their hands on the keyboard. (3) The program will automatically and randomly present standard and target stimuli. (4) E−Prime 3.0 software and the EEG recorder will be activated to synchronously record stimulus markers, EEG signals, and button−press responses. (5) The acquisition will last approximately 3 minutes. The specific operational procedure is shown in **Figure 3**.

### Safety outcomes

Safety and tolerability will be systematically assessed throughout the study. Treatment-emergent adverse events will be monitored using the TESS, a standardized clinician-rated instrument for evaluating the presence, frequency, and severity of stimulation-related side effects. Adverse events of interest include, but are not limited to, headache, dizziness, nausea, scalp discomfort, muscle twitching, and other potential symptoms associated with transcranial magnetic stimulation. All adverse events will be recorded at each treatment session and follow-up visit, and their severity and potential relationship to the intervention will be documented. For further details, please see **Supplementary Material S3**.

### Statistical analysis

#### Sample size

The sample size for this exploratory trial was determined *a priori* using G*Power software (version 3.1). An F-test was employed based on an effect size (Cohen’s d) of 0.38 by Chu et al. (2021). A total of 50 participants were required to achieve 80% statistical power at a two-sided alpha level of 0.05 (37). Anticipating an approximate dropout rate of 10%, the planned enrollment was at 56 participants.

#### Analysis of clinical data

All statistical analyses will be conducted using SPSS version 26.0. Continuous variables will be presented as means ± standard deviations or medians (interquartile ranges), depending on distribution, and compared using independent samples t-tests or Mann-Whitney U tests, as appropriate. Categorical variables will be expressed as frequencies (%) and analyzed using Chi-square or Fisher’s exact tests. A repeated-measures analysis of variance (ANOVA) will be applied to evaluate longitudinal changes in each outcome, with group (active vs sham) as the between-subject factor and time (baseline, post-intervention, follow-up) as the within-subject factor. If the assumption of sphericity is violated according to Mauchly’s test, the Greenhouse-Geisser correction will be applied. Significant interactions will be followed by Bonferroni-adjusted simple-effects comparisons to determine between-group differences at each time point and within-group changes across time.

### Analysis of EEG

#### Data preprocessing (using EEGLAB software)

EEG preprocessing will be performed using the EEGLAB toolbox in MATLAB, comprising filtering, artifact removal, epoching, baseline correction, and event-related potential (ERP) averaging (38). Continuous data will first be band-pass filtered (0.1–0.5 Hz high-pass; 30–40 Hz low-pass) to attenuate slow drifts, electromyographic noise, and line interference. Independent Component Analysis will then be applied to remove ocular and myogenic artifacts, with components identified based on spatial topography and time-frequency characteristics. Data will subsequently be segmented into stimulus-locked epochs and baseline-corrected using a pre-stimulus interval to minimize low-frequency fluctuations. Artifact-free epochs for target and standard tones will be averaged separately to enhance event-related activity and suppress background EEG. Finally, the P300 component will be identified as the most positive peak within the 300–600 ms post-stimulus window at the Cz electrode, with both baseline-to-peak amplitude and peak latency quantified for subsequent statistical modeling.

#### Adverse events

All adverse events (AEs) will be systematically monitored and documented throughout the trial. For each AE, the occurrence time, severity, duration, clinical management, and outcome will be recorded, along with the investigator’s judgment of its relationship to the intervention. Serious Adverse Events (SAEs) will be defined as events resulting in death, life-threatening conditions, hospitalization or prolonged hospitalization, or significant functional disability. Any SAE will be reported immediately to the institutional ethics committee in accordance with regulatory requirements.

Given the established safety profile of transcranial magnetic stimulation, expected AEs include mild and transient symptoms such as scalp discomfort, headache, facial muscle twitching, dizziness, or fatigue. Rare but potential risks (e.g., induction of seizure) will be communicated to participants during the informed-consent process (39). If any AE occurs, appropriate clinical management will be provided, and participants will be followed until symptom resolution or stabilization.

### Ethics and results dissemination

This study complies with the Declaration of Helsinki and has received approval from the Ethics Committee of Wuxi Mental Health Center (Approval No.: WXMHCIRB2025LLky101). The trial is registered with the clinical trial registry (Registration No.: ChiCTR2500109181). All participants will provide written informed consent after receiving clear information about the study. Participation is voluntary, and individuals may withdraw at any time without consequences. Study findings will be disseminated through peer-reviewed publications and scientific conferences.

### Data management

Study data will be recorded in the Case Report Form (CRF) in a timely, comprehensive, and accurate manner. Two researchers will separately enter the data into EpiData software (Version 3.1, developed by JM Lauritsen of the EpiData Association) (40). Subsequently, the built-in double-check function of the software will be used to verify the entered data, ensuring data accuracy. All study-related electronic files will only be accessible to authorized personnel, and computer systems will adopt password protection measures to ensure data security. To ensure the consistency of study-related documents, each participant will be assigned a unique identification number upon enrollment. Paper-based CRFs and study documents will be stored in a designated secure office area, managed by the study coordinator, and properly kept in a locked security cabinet.

Researchers must strictly adhere to the study protocol, conduct timely verification, collection, recording, and protection of data, and minimize the possibility of data loss. If data are missing for a small number of patients, multiple imputation will be used for processing. Meanwhile, source data verification will be conducted by comparing registered data with original medical records to assess the accuracy, completeness, and representativeness of the registered data.

### Data monitoring committee

Given the study’s single-center design, moderate sample size, short intervention duration, and the well-established low-risk profile of TMS-based interventions, a formal independent DMC has not been established for this trial. All SAEs will be reported to the Ethics Committee of Wuxi Mental Health Center within 24 hours of confirmation, in strict compliance with local regulatory requirements and the Declaration of Helsinki.

### Discontinuation of assigned intervention

Participants may withdraw from the study at any time without penalty. The following discontinuation criteria apply throughout the entire study period. The Principal Investigator may discontinue the assigned intervention if any of the following occur:

1. Seizure or other acute neurological event.
2. Emergent mania meeting DSM-5 criteria.
3. New-onset psychotic symptoms.
4. Persistent non-adherence to study procedures.
5. Illicit substance use or a positive urine toxicology test.

Participants discontinued from the intervention will continue to receive standard clinical care, and their safety will be monitored until symptoms resolve or stabilize.

### Confidentiality

Participating investigators and their research staff will strictly maintain participant confidentiality and privacy. Accordingly, all study-related documents, data, and other generated materials will be treated with strict confidentiality. No study-related information or data will be disclosed to unauthorized third parties without prior written approval. All study procedures will be conducted in environments ensuring maximum privacy protection. Potential participants will be offered the opportunity to discuss study involvement without parents or guardians present.

All participant information will be managed in compliance with institutional and regulatory data protection standards. Personally identifiable data will be replaced with unique study identification codes, with access to the encryption key restricted to authorized research personnel. Electronic data will be stored on password-protected systems, and physical records will be secured in locked cabinets within controlled-access research facilities. No identifiable information will appear in any reports, presentations, or publications.

## Discussion

This study addresses the urgent clinical need for rapid and safe interventions targeting suicidal ideation in adults with MDD. Sequential bilateral aTBS integrates inhibitory and excitatory stimulation within a condensed treatment schedule, offering a potential balance between therapeutic efficiency and tolerability. To our knowledge, this represents the first randomized controlled trial specifically designed to systematically evaluate the efficacy and safety of sequential aTBS in adults presenting with suicidal ideation.

This trial has several notable strengths. First, as the first randomized, single-blind, sham-controlled study specifically designed to evaluate sequential accelerated theta-burst stimulation in adults with major depressive disorder accompanied by suicidal ideation, this work directly addresses a critical evidence gap in the field of neuromodulation. Second, the protocol integrates a bilateral sequential stimulation approach—right-cTBS followed by left-iTBS—that is theoretically grounded in contemporary models of interhemispheric imbalance and network dysfunction in depression (41, 42). This design allows simultaneous engagement of inhibitory and excitatory prefrontal pathways within a single session, potentially enhancing the efficiency of network-level modulation relative to single-target TBS paradigms. Third, by employing an accelerated schedule (10 sessions across 10 weekdays), the study evaluates a clinically pragmatic, time-efficient treatment model that may be particularly valuable for patients presenting with elevated suicide risk who require rapid intervention. Fourth, the inclusion of electrophysiological measures (EEG-derived P300) provides an important mechanistic dimension, enabling the assessment of neurophysiological changes associated with aTBS and offering potential biomarkers of treatment response. Collectively, these methodological features strengthen both the clinical relevance and mechanistic interpretability of the trial.

Several limitations of this study must be acknowledged. This is a single-center investigation with a modest sample size. The single-blind design employed in this study may introduce potential investigator bias. Regarding the limitations of the sham stimulation method, this study employs the coil tilting method as the sham control condition. Although this method is widely used, it has limitations, including incomplete matching of somatosensory experience and lack of operator blinding, which may potentially impact participant blinding. To assess the extent of blinding compromise, we will conduct a blinding integrity check after the intervention and incorporate sensitivity analyses into the statistical analysis to examine the robustness of the results. Future studies should adopt dedicated sham coils and double-blind designs to further reduce the risk of bias. The mechanistic analysis based on EEG is exploratory in nature and cannot establish causal inference. Nonetheless, these design choices reflect a balance between feasibility and scientific rigor, providing essential preliminary data to inform future multicenter trials. In conclusion, this study is expected to yield valuable clinical and safety evidence for sequential aTBS as a rapid and well-tolerated intervention for suicidal ideation in adults with MDD.
