# Supplementary material for: Clinical efficacy and safety of sequential accelerated theta burst stimulation for suicidal ideation in adults with major depressive disorder: study protocol of a randomized controlled trial
Source: Front Psychiatry. 2026 Mar 31;17:1795183. doi: 10.3389/fpsyt.2026.1795183 (PMC13076289; doi:10.3389/fpsyt.2026.1795183)
Supplement: Supplementary file 1 [file DataSheet1.docx]

**Supplementary Material**

**Table of content**

目录

[Supplementary material S1. SPIRIT 2025 2](#_Toc31339)

[Supplementary material S2. Informed Consent Form (ICF) 6](#_Toc13261)

[Supplementary material S3. Treatment Emergent Symptom Scale (TESS) 9](#_Toc16327)

# Supplementary material S1. SPIRIT 2025

| **Section / Topic** | **No** | **SPIRIT 2025 checklist item description** | **Reported on page no.** |
| --- | --- | --- | --- |
| **Administrative information** | | |  |
| Title and structured summary | 1a | Title stating the trial design, population, and interventions, with identification as a protocol | 1 |
|  | 1b | Structured summary of trial design and methods, including items from the World Health Organization Trial Registration Data Set | 2 |
| Protocol version | 2 | Version date and identifier | 6-7 |
| Roles and responsibilities | 3a | Names, affiliations, and roles of protocol contributors | 23 |
|  | 3b | Name and contact information for the trial sponsor | 1 |
|  | 3c | Role of trial sponsor and funders in design, conduct, analysis, and reporting of trial; including any authority over these activities | 23,24 |
|  | 3d | Composition, roles, and responsibilities of the coordinating site, steering committee, endpoint adjudication committee, data management team, and other individuals or groups overseeing the trial, if applicable | 20-23 |
| **Open science** | | |  |
| Trial registration | 4 | Name of trial registry, identifying number (with URL), and date of registration. If not yet registered, name of intended registry | 2 |
| Protocol and statistical analysis plan | 5 | Where the trial protocol and statistical analysis plan can be accessed | 6,7 |
| Data sharing | 6 | Where and how the individual de-identified participant data (including data dictionary), statistical code, and any other materials will be accessible | 20-21 |
| Funding and conflicts of interest | 7a | Sources of funding and other support (e.g., supply of drugs) | 24 |
|  | 7b | Financial and other conflicts of interest for principal investigators and steering committee members | 24 |
| Dissemination policy | 8 | Plans to communicate trial results to participants, healthcare professionals, the public, and other relevant groups (e.g., reporting in trial registry, plain language summary, publication) | 24 |
| **Introduction** | | |  |
| Background and rationale | 9a | Scientific background and rationale, including summary of relevant studies (published and unpublished) examining benefits and harms for each intervention | 3-6 |
|  | 9b | Explanation for choice of comparator | 4-6 |
| Objectives | 10 | Specific objectives related to benefits and harms | 6 |
| **Methods: Patient and public involvement, trial design** | | |  |
| Patient and public involvement | 11 | Details of, or plans for, patient or public involvement in the design, conduct, and reporting of the trial | 24 |
| Trial design | 12 | Description of trial design including type of trial (e.g., parallel group, crossover), allocation ratio, and framework (e.g., superiority, equivalence, non-inferiority, exploratory) | 6,7 |
| **Methods: Participants, interventions, and outcomes** | | |  |
| Trial setting | 13 | Settings (e.g., community, hospital) and locations (e.g., countries, sites) where the trial will be conducted | 7 |
| Eligibility criteria | 14a | Eligibility criteria for participants | 9-10 |
|  | 14b | If applicable, eligibility criteria for sites and for individuals who will deliver the interventions (e.g., surgeons, physiotherapists) | 6-7 |
| Intervention and comparator | 15a | Intervention and comparator with sufficient details to allow replication including how, when, and by whom they will be administered. If relevant, where additional materials describing the intervention and comparator (e.g., intervention manual) can be accessed | 7-8,11-14 |
|  | 15b | Criteria for discontinuing or modifying allocated intervention/comparator for a trial participant (e.g., drug dose change in response to harms, participant request, or improving/worsening disease) | 20 |
|  | 15c | Strategies to improve adherence to intervention/comparator protocols, if applicable, and any procedures for monitoring adherence (e.g., drug tablet return, sessions attended) | 13 |
|  | 15d | Concomitant care that is permitted or prohibited during the trial | 13 |
| Outcomes | 16 | Primary and secondary outcomes, including the specific measurement variable (e.g., systolic blood pressure), analysis metric (e.g., change from baseline, final value, time to event), method of aggregation (e.g., median, proportion), and time point for each outcome | 14-15 |
| Harms | 17 | How harms are defined and will be assessed (e.g., systematically, non-systematically) | 17-18 |
| Participant timeline | 18 | Time schedule of enrollment, interventions (including any run-ins and washouts), assessments, and visits for participants. A schematic diagram is highly recommended (see Figure) | 9 |
| Sample size | 19 | How sample size was determined, including all assumptions supporting the sample size calculation | 18-19 |
| Recruitment | 20 | Strategies for achieving adequate participant enrollment to reach target sample size | 18 |
| **Methods: Assignment of interventions** | | |  |
| Randomization: |  |  |  |
| Sequence generation | 21a | Who will generate the random allocation sequence and the method used | 11-12 |
|  | 21b | Type of randomization (simple or restricted) and details of any factors for stratification. To reduce predictability of a random sequence, other details of any planned restriction (e.g., blocking) should be provided in a separate document that is unavailable to those who enroll participants or assign interventions | 11-12 |
| Allocation concealment  mechanism | 22 | Mechanism used to implement the random allocation sequence (e.g., central computer/telephone; sequentially numbered, opaque, sealed containers), describing any steps to conceal the sequence until interventions are assigned | 11-12 |
| Implementation | 23 | Whether the personnel who will enroll and those who will assign participants to the interventions will have access to the random allocation sequence | 11-12 |
| Blinding | 24a | Who will be blinded after assignment to interventions (e.g., participants, care providers, outcome assessors, data analysts) | 12 |
|  | 24b | If blinded, how blinding will be achieved and description of the similarity of interventions | 12 |
|  | 24c | If blinded, circumstances under which unblinding is permissible, and procedure for revealing a participant’s allocated intervention during the trial | 12 |
| **Methods: Data collection, management, and analysis** | | |  |
| Data collection methods | 25a | Plans for assessment and collection of trial data, including any related processes to promote data quality (e.g., duplicate measurements, training of assessors) and a description of trial instruments (e.g., questionnaires, laboratory tests) along with their reliability and validity, if known. Reference to where data collection forms can be accessed, if not in the protocol | 14-17 |
|  | 25b | Plans to promote participant retention and complete follow-up, including list of any outcome data to be collected for participants who discontinue or deviate from intervention protocols | 19-20 |
| Data management | 26 | Plans for data entry, coding, security, and storage, including any related processes to promote data quality (e.g., double data entry; range checks for data values). Reference to where details of data management procedures can be accessed, if not in the protocol | 20-21 |
| Statistical methods | 27a | Statistical methods used to compare groups for primary and secondary outcomes, including harms | 18-19 |
|  | 27b | Definition of who will be included in each analysis (e.g., all randomized participants), and in which group | 18-19 |
|  | 27c | How missing data will be handled in the analysis | 21 |
|  | 27d | Methods for any additional analyses (e.g., subgroup and sensitivity analyses) | N/A |
| **Methods: Monitoring** | | |  |
| Data monitoring committee | 28a | Composition of data monitoring committee (DMC); summary of its role and reporting structure; statement of whether it is independent from the sponsor and funder; conflicts of interest and reference to where further details about its charter can be found, if not in the protocol. Alternatively, an explanation of why a DMC is not needed | 21 |
|  | 28b | Explanation of any interim analyses and stopping guidelines, including who will have access to these interim results and make the final decision to terminate the trial | 21 |
| Trial monitoring | 29 | Frequency and procedures for monitoring trial conduct. If there is no monitoring, give explanation | 19-21 |
| **Ethics** | | |  |
| Research ethics approval | 30 | Plans for seeking research ethics committee/institutional review board approval | 20 |
| Protocol amendments | 31 | Plans for communicating important protocol modifications to relevant parties | 20 |
| Consent or assent | 32a | Who will obtain informed consent or assent from potential trial participants or authorized proxies, and how | 20 |
|  | 32b | Additional consent provisions for collection and use of participant data and biological specimens in ancillary studies, if applicable | N/A |
| Confidentiality | 33 | How personal information about potential and enrolled participants will be collected, shared, and maintained in order to protect confidentiality before, during, and after the trial | 20-22 |
| Ancillary and post-trial care | 34 | Provisions, if any, for ancillary and post-trial care, and for compensation to those who suffer harm from trial participation | 24-25 |

# Supplementary material S2. Informed Consent Form(ICF)

**Dear Sir/Madam,**

We invite you to participate in a research project entitled Efficacy and Neurophysiological Mechanisms of Sequential aTBS for Suicidal Ideation in Adults with Major Depressive Disorder. This project is a research program of the Affiliated Mental Health Center of Jiangnan University and has been approved by the Ethics Committee of Wuxi Mental Health Center.

**Research Objectives**

Primary Objective

The primary aim of this randomized controlled trial is to evaluate the clinical efficacy of sequential accelerated Theta Burst Stimulation (aTBS) in adult patients with Major Depressive Disorder (MDD).

Secondary Objectives

To systematically assess the safety, tolerability, and adverse event profile of sequential aTBS in the target population.

Study Procedures

This study adopts a randomized, single-blind controlled experimental design. General demographic data will be collected, scale assessments will be conducted, and biological samples will be obtained. The improvement of suicidal ideation and depressive symptoms before and after sequential aTBS treatment will be analyzed. Cognitive function will be evaluated using depression and suicidal ideation scales, as well as P300 testing, at the patient’s baseline and after 10 days of intervention, and the correlation between cognitive function and the improvement of depressive symptoms will be analyzed.

**Risk Disclosure**

If you decide to participate in this study, your trial participation and personal data collected during the trial will be kept confidential. The attending researchers and other study staff will use your medical information for research purposes. Such information may include your name, address, telephone number, medical history, and data obtained during your study visits. Your behavioral and brain imaging data will be identified by study-specific coded numbers instead of your name. Information that can identify your identity will not be disclosed to individuals outside the research team unless we obtain your written permission. No personal information about you will be disclosed when the results of this study are published.

**Benefits to Participants**

You will not receive any direct benefits from participating in this study. However, you are not required to bear the costs of any assessments and tests involved in the study during the intervention period. The research team will provide corresponding assessment and test reports for your reference. For participants who may have been discharged from the hospital at the start of the study, a transportation allowance of 50 RMB will be provided for each hospital visit.

**Participation Principles**

This study adheres to the principle of voluntary participation. You may refuse to participate or withdraw from the study at any time during the research process. Your decision will not affect your future medical treatment. If you decide to withdraw from the study, please notify the researchers in advance. If the researchers assess that you are no longer suitable to continue participating in the study during the trial, they also have the right to request your withdrawal. If you have any questions about this study, you may call the Ethics Committee office at 83219310 for consultation and resolution.

Participant Statement

I have read the above introduction to the study and fully understand the potential risks and benefits of participating in this research. I voluntarily consent to participate in the clinical study described herein.

Signature of Participant ________________________ Date _____________________

Guardian’s Signature (if applicable)_____________ Date _____________________

Researcher's Statement

I confirm that I have fully explained the details of this research project to the participant, particularly the potential risks and benefits associated with participation.

Researcher's Signature: _________________________ Date_________________________

# Supplementary material S3. Treatment Emergent Symptom Scale (TESS)

**Treatment Emergent Symptom Scale (TESS)**

Name_________ Gender____ Age____

Instructions: This questionnaire is designed for psychiatrists to assess side effects caused by various psychotropic medications in adult patients. Please rate based on patient reports, physical examination results, and laboratory reports. For some items, inquiries should also be made with the patient's family or ward staff. Assessment times: before treatment, and at 2 weeks and 4 weeks after treatment.

**1.Toxic Confusion:** 1) Absent 2) Minimal or doubtful 3) Mild 4) Occurs only at night, brief 5) Persists into the daytime.
**Management:** 1) None 2) Increased observation 3) Administer antidote 4) Reduce dose 5) Reduce dose and administer antidote 6) Suspend treatment 7) Discontinue treatment

**2.Excitement/Agitation:** 1) Absent 2) Minimal or doubtful 3) Anxiety or fear present 4) Non-persistent agitated motor behavior 5) Persistent agitation, e.g., head-banging, foot-stamping, hand-wringing.
**Management:** 1) None 2) Increased observation 3) Administer antidote 4) Reduce dose 5) Reduce dose and administer antidote 6) Suspend treatment 7) Discontinue treatment

**3.Depressed Mood:** 1) Absent 2) Minimal or doubtful 3) Depressed mood elicited on questioning 4) Spontaneous reports of depression, hopelessness, tearfulness 5) Major depressive episode meeting diagnostic criteria, with retardation.
**Management:** 1) None 2) Increased observation 3) Administer antidote 4) Reduce dose 5) Reduce dose and administer antidote 6) Suspend treatment 7) Discontinue treatment

**4.Increased Activity:** 1) Absent 2) Minimal or doubtful 3) Non-persistent, can be self-controlled 4) Persistent, does not require external control 5) Persistent, requires others' intervention.
**Management:** 1) None 2) Increased observation 3) Administer antidote 4) Reduce dose 5) Reduce dose and administer antidote 6) Suspend treatment 7) Discontinue treatment

**5.Decreased Activity:** 1) Absent 2) Minimal or doubtful 3) Spontaneous activity reduced 4) Requires prompting to be active 5) Stupor or sub-stupor.
**Management:** 1) None 2) Increased observation 3) Administer antidote 4) Reduce dose 5) Reduce dose and administer antidote 6) Suspend treatment 7) Discontinue treatment

**6.Insomnia:** 1) Absent 2) Minimal or doubtful 3) Sleep reduced by 2 hours compared to usual 4) Reduced by 3-6 hours 5) Reduced by more than 6 hours.
**Management:** 1) None 2) Increased observation 3) Administer antidote 4) Reduce dose 5) Reduce dose and administer antidote 6) Suspend treatment 7) Discontinue treatment

**7.Somnolence (Drowsiness):** 1) Absent 2) Minimal or doubtful 3) Daytime drowsiness or naps for 2 hours 4) Daytime sleep for 3-8 hours 5) Daytime sleep for more than 8 hours.
**Management:** 1) None 2) Increased observation 3) Administer antidote 4) Reduce dose 5) Reduce dose and administer antidote 6) Suspend treatment 7) Discontinue treatment

**8.Hematological Abnormalities:** 1) Absent 2) Minimal or doubtful 3) Mild 4) Abnormal lab results, e.g., leukopenia 5) Severe abnormality, e.g., agranulocytosis.
**Management:** 1) None 2) Increased observation 3) Administer antidote 4) Reduce dose 5) Reduce dose and administer antidote 6) Suspend treatment 7) Discontinue treatment

**9.Liver Function:** 1) Absent 2) Minimal or doubtful 3) Mild 4) Abnormal lab results 5) Jaundice.
**Management:** 1) None 2) Increased observation 3) Administer antidote 4) Reduce dose 5) Reduce dose and administer antidote 6) Suspend treatment 7) Discontinue treatment

**10.Urinalysis Abnormalities:** 1) Absent 2) Minimal or doubtful 3) Mild 4) Definitely abnormal lab results 5) Severely abnormal.
**Management:** 1) None 2) Increased observation 3) Administer antidote 4) Reduce dose 5) Reduce dose and administer antidote 6) Suspend treatment 7) Discontinue treatment

**11.Muscle Rigidity:** 1) Absent 2) Minimal or doubtful 3) Slight increase in muscle tone, not affecting movement 4) Marked increase in muscle tone (without antidote use) 5) Extreme muscle rigidity, not reversible even with antidote.
**Management:** 1) None 2) Increased observation 3) Administer antidote 4) Reduce dose 5) Reduce dose and administer antidote 6) Suspend treatment 7) Discontinue treatment

**12.Tremor:** 1) Absent 2) Minimal or doubtful 3) Subjective feeling of tremor, or mild tremor with eyes closed and hands extended 4) Visible tremor, affecting fine activities 5) Severe tremor, affecting daily life, e.g., unable to eat.
**Management:** 1) None 2) Increased observation 3) Administer antidote 4) Reduce dose 5) Reduce dose and administer antidote 6) Suspend treatment 7) Discontinue treatment

**13.Dystonia:** 1) Absent 2) Minimal or doubtful 3) Present, but does not affect activity 4) Affects activity but not daily life 5) Affects daily life.
**Management:** 1) None 2) Increased observation 3) Administer antidote 4) Reduce dose 5) Reduce dose and administer antidote 6) Suspend treatment 7) Discontinue treatment

**14.Akathisia:** 1) Absent 2) Minimal or doubtful 3) Subjective restlessness, impatience, can self-control 4) Due to impatience, stands up or walks during interview or work 5) Unable to sit still, unable to complete tasks, loss of self-control.
**Management:** 1) None 2) Increased observation 3) Administer antidote 4) Reduce dose 5) Reduce dose and administer antidote 6) Suspend treatment 7) Discontinue treatment

**15.Dry Mouth:** 1) Absent 2) Minimal or doubtful 3) Complains of dry oral mucosa 4) Detectable dry oral mucosa (no significant impact on life) 5) Clearly detectable dry oral mucosa (severely affects patient's activities and life).
**Management:** 1) None 2) Increased observation 3) Administer antidote 4) Reduce dose 5) Reduce dose and administer antidote 6) Suspend treatment 7) Discontinue treatment

**16.Nasal Congestion:** 1) Absent 2) Minimal or doubtful 3) Subjective feeling of congestion 4) Observable or verifiable congestion (e.g., voice change) (no significant impact on life) 5) Observable or verifiable congestion (severely affects patient's activities and life).
**Management:** 1) None 2) Increased observation 3) Administer antidote 4) Reduce dose 5) Reduce dose and administer antidote 6) Suspend treatment 7) Discontinue treatment

**17.Blurred Vision:** 1) Absent 2) Minimal or doubtful 3) Subjective complaint only 4) Affects visual acuity 5) Interferes with daily activities, e.g., bumping into objects.
**Management:** 1) None 2) Increased observation 3) Administer antidote 4) Reduce dose 5) Reduce dose and administer antidote 6) Suspend treatment 7) Discontinue treatment

**18.Constipation:** 1) Absent 2) Minimal or doubtful 3) Constipation for over 36 hours 4) Constipation for over 4 days 5) Requires manual disimpaction.
**Management:** 1) None 2) Increased observation 3) Administer antidote 4) Reduce dose 5) Reduce dose and administer antidote 6) Suspend treatment 7) Discontinue treatment

**19.Increased Salivation:** 1) Absent 2) Minimal or doubtful 3) Mild 4) Increased saliva 5) Drooling.
**Management:** 1) None 2) Increased observation 3) Administer antidote 4) Reduce dose 5) Reduce dose and administer antidote 6) Suspend treatment 7) Discontinue treatment

**20.Sweating:** 1) Absent 2) Minimal or doubtful 3) Sweat more than usual, or episodes of sweating 4) Sweat more than usual, or episodes of sweating (some impact on life) 5) Profuse facial sweating.
**Management:** 1) None 2) Increased observation 3) Administer antidote 4) Reduce dose 5) Reduce dose and administer antidote 6) Suspend treatment 7) Discontinue treatment

**21.Nausea/Vomiting:** 1) Absent 2) Minimal or doubtful 3) Mild 4) Nausea 5) Vomiting.
**Management:** 1) None 2) Increased observation 3) Administer antidote 4) Reduce dose 5) Reduce dose and administer antidote 6) Suspend treatment 7) Discontinue treatment

**22.Diarrhea:** 1) Absent 2) Minimal or doubtful 3) Twice a day 4) 3-5 times a day 5) More than 5 times a day.
**Management:** 1) None 2) Increased observation 3) Administer antidote 4) Reduce dose 5) Reduce dose and administer antidote 6) Suspend treatment 7) Discontinue treatment

**23.Hypotension:** 1) Absent 2) Minimal or doubtful 3) Mild 4) >10% lower than usual 5) >20% lower than usual or too low to measure.
**Management:** 1) None 2) Increased observation 3) Administer antidote 4) Reduce dose 5) Reduce dose and administer antidote 6) Suspend treatment 7) Discontinue treatment

**24.Dizziness/Lightheadedness:** 1) Absent 2) Minimal or doubtful 3) Feeling dizzy/lightheaded 4) Dizziness/lightheadedness with loss of balance 5) Fainting, loss of consciousness.
**Management:** 1) None 2) Increased observation 3) Administer antidote 4) Reduce dose 5) Reduce dose and administer antidote 6) Suspend treatment 7) Discontinue treatment

**25.Tachycardia:** (Measured upon morning waking) 1) Absent 2) Minimal or doubtful 3) Heart rate 90-100 bpm 4) 100-120 bpm 5) >120 bpm.
**Management:** 1) None 2) Increased observation 3) Administer antidote 4) Reduce dose 5) Reduce dose and administer antidote 6) Suspend treatment 7) Discontinue treatment

**26.Hypertension:** (For patients without hypertension before treatment) 1) Absent 2) Minimal or doubtful 3) >140/90 mmHg 4) >160/100 mmHg 5) >200/120 mmHg.
**Management:** 1) None 2) Increased observation 3) Administer antidote 4) Reduce dose 5) Reduce dose and administer antidote 6) Suspend treatment 7) Discontinue treatment

**27.ECG Abnormalities:** 1) Absent 2) Minimal or doubtful 3) Abnormal, but not clinically significant 4) Clinically significant abnormality 5) Abnormality with serious consequences.
**Management:** 1) None 2) Increased observation 3) Administer antidote 4) Reduce dose 5) Reduce dose and administer antidote 6) Suspend treatment 7) Discontinue treatment

**28.Skin Symptoms:** 1) Absent 2) Minimal or doubtful 3) Photosensitivity 4) Transient itching or erythema 5) Allergic dermatitis.
**Management:** 1) None 2) Increased observation 3) Administer antidote 4) Reduce dose 5) Reduce dose and administer antidote 6) Suspend treatment 7) Discontinue treatment

**29.Weight Gain:** 1) Absent 2) Minimal or doubtful 3) Gain of 5 lbs (~2.3 kg) in one month 4) Gain of 6-10 lbs (~2.7-4.5 kg) 5) Gain of >10 lbs (~4.5 kg).
**Management:** 1) None 2) Increased observation 3) Administer antidote 4) Reduce dose 5) Reduce dose and administer antidote 6) Suspend treatment 7) Discontinue treatment

**30.Weight Loss:** 1) Absent 2) Minimal or doubtful 3) Loss of 5 lbs (~2.3 kg) in one month 4) Loss of 6-10 lbs (~2.7-4.5 kg) 5) Loss of >10 lbs (~4.5 kg).
**Management:** 1) None 2) Increased observation 3) Administer antidote 4) Reduce dose 5) Reduce dose and administer antidote 6) Suspend treatment 7) Discontinue treatment

**31.Decreased Appetite/Anorexia:** 1) Absent 2) Minimal or doubtful 3) Daily food intake equivalent to only two meals 4) Equivalent to one meal 5) No food intake.
**Management:** 1) None 2) Increased observation 3) Administer antidote 4) Reduce dose 5) Reduce dose and administer antidote 6) Suspend treatment 7) Discontinue treatment

**32.Headache:** 1) Absent 2) Minimal or doubtful 3) Subjective complaint only 4) Painful 5) Leading to loss of function or incapacitation.
**Management:** 1) None 2) Increased observation 3) Administer antidote 4) Reduce dose 5) Reduce dose and administer antidote 6) Suspend treatment 7) Discontinue treatment

**33.Tardive Dyskinesia (TD) Severity:** 1) Absent 2) Minimal or doubtful 3) TD symptoms elicited on examination 4) Spontaneous TD symptoms 5) Significantly affects function or activity.
**Management:** 1) None 2) Increased observation 3) Administer antidote 4) Reduce dose 5) Reduce dose and administer antidote 6) Suspend treatment 7) Discontinue treatment

**34.Other ______** (To be specified by rater) **Severity:** 1) Absent 2) Minimal or doubtful 3) Mild 4) Moderate 5) Severe.
**Management:** 1) None 2) Increased observation 3) Administer antidote 4) Reduce dose 5) Reduce dose and administer antidote 6) Suspend treatment 7) Discontinue treatment

**35.Other ______** (To be specified by rater) **Severity:** 1) Absent 2) Minimal or doubtful 3) Mild 4) Moderate 5) Severe.
**Management:** 1) None 2) Increased observation 3) Administer antidote 4) Reduce dose 5) Reduce dose and administer antidote 6) Suspend treatment 7) Discontinue treatment

**36.Global Assessment** (Not required before treatment)

**A.** Compared to other patients in this study, the severity of treatment-emergent side effects is:
1=None 2=Mild 3=Moderate 4=Severe 5=Uncertain

**B.** Compared to other patients in this study, the patient's reported distress due to side effects is:
1=None 2=Mild 3=Moderate 4=Severe 5=Uncertain
